# Gla-Rich Protein (GRP): A Vitamin K-Dependent Regulator of Vascular Calcification, Inflammation, and Mineral Homeostasis

**DOI:** 10.3390/cimb48050458

**Published:** 2026-04-29

**Authors:** Antun Loncaric, Lara Baticic

**Affiliations:** 1 Department of Cardiology, General Hospital Dr. Ivo Pedisic Sisak, 44000 Sisak, Croatia; antun.loncaric@uniri.hr; 2 Department of Medical Chemistry, Biochemistry and Clinical Chemistry, Faculty of Medicine, University of Rijeka, 51000 Rijeka, Croatia

**Keywords:** chronic kidney disease, Gla-rich protein, mineral homeostasis, inflammation, vitamin K, vascular calcification

## Abstract

Gla-rich protein (GRP), also known as UCMA, is a vitamin K-dependent protein that has emerged as an important regulator of pathological calcification and inflammation. Vascular calcification is a major complication of chronic kidney disease and cardiovascular disorders and is now recognized as an active and tightly regulated process rather than a passive accumulation of minerals. Increasing evidence indicates that GRP plays a protective role in mineral homeostasis through its strong calcium-binding capacity and its dependence on vitamin K-mediated gamma carboxylation. This work represents a comprehensive narrative review aimed at summarizing and critically discussing the current scientific knowledge on GRP. Available experimental and clinical data are analyzed with respect to gene expression, molecular regulation, vitamin K dependency, and underlying mechanisms of action. Particular emphasis is placed on the dual function of GRP in inhibiting ectopic calcification and modulating inflammatory responses. The evidence linking altered GRP levels or changes in its carboxylation status with chronic kidney disease, vascular calcification, calcific aortic valve disease, osteoarthritis, and tumor-associated microcalcifications is systematically examined. Current findings collectively support the concept that GRP is a multifunctional protein operating at the interface of mineral metabolism, inflammation, and tissue remodeling. Despite promising experimental data, important knowledge gaps remain, including the absence of standardized assays capable of distinguishing different GRP forms and the lack of longitudinal clinical studies evaluating its predictive value. This manuscript highlights the potential of GRP as a biomarker of disturbed mineral homeostasis and cardiovascular risk, while emphasizing the need for further research to clarify its precise biological functions and clinical relevance.

## 1. Introduction

Vascular calcification (VC) is defined as the pathological deposition of calcium–phosphate mineral complexes, predominantly hydroxyapatite, within the vascular wall, and is now recognized as a highly regulated, cell-mediated process rather than a passive degenerative phenomenon [1,2,3,4,5]. Depending on the affected vascular compartment, calcification may develop within the intimal layer, where it is commonly associated with atherosclerotic plaque formation, or within the medial layer, where it occurs largely independently of lipid accumulation and primarily contributes to arterial stiffening [1,2,6]. In both forms, mineral deposition disrupts vascular architecture, reduces arterial elasticity, increases pulse wave velocity, and contributes to elevated systolic blood pressure, widened pulse pressure, left ventricular hypertrophy, and impaired coronary perfusion, thereby increasing cardiovascular risk [1,2,3]. More broadly, VC should be viewed within the framework of ectopic biomineralization, in which pathological mineral deposition occurs in soft tissues that normally remain non-mineralized [1,7]. This concept is clinically important because pathological calcification reflects not only mineral excess, but also a disturbance in the balance between pro-calcific stimuli and endogenous inhibitory systems [1,3,8].

VC is particularly relevant in chronic kidney disease (CKD), where cardiovascular disease remains the leading cause of morbidity and mortality and where calcification represents one of the most important non-traditional cardiovascular risk factors [3,9,10]. CKD affects more than 10% of the global population, with recent estimates indicating that nearly 788 million people worldwide may be affected [11,12]. As kidney function declines, abnormalities in phosphate and calcium balance, parathyroid hormone regulation, vitamin D metabolism, fibroblast growth factor 23 (FGF-23), and Klotho signaling converge within the syndrome of CKD–mineral and bone disorder (CKD-MBD), creating a systemic pro-calcific milieu [5,9,10,13,14]. This altered milieu promotes osteogenic transdifferentiation of vascular smooth muscle cells (VSMCs), extracellular vesicle release, calciprotein particle maturation, and progressive mineral deposition within the vascular wall [2,3,15,16,17]. Current evidence indicates that VC is driven by a complex interplay among phosphate overload, inflammation, oxidative stress, apoptosis, extracellular matrix remodeling, and osteogenic signaling pathways, rather than by passive calcium precipitation alone [2,3,18,19]. Under calcifying conditions, VSMCs lose their contractile phenotype and acquire osteogenic, chondrogenic, or osteocyte-like features, accompanied by expression of bone-related transcription factors and matrix proteins [2,3,20]. In physiological conditions, several endogenous inhibitors protect soft tissues from inappropriate mineralization, including matrix Gla protein (MGP), fetuin-A, osteopontin (OPN), osteoprotegerin (OPG), and Gla-rich protein (GRP) [4,7,8,21]. Among these inhibitors, vitamin K-dependent proteins (VKDPs) are of particular importance because their biological function depends on γ-carboxylation of specific glutamate residues to γ-carboxyglutamate (Gla), a post-translational modification required for efficient calcium binding and mineral interaction [22,23,24]. Accordingly, inadequate vitamin K availability or impaired γ-carboxylation leads to the accumulation of undercarboxylated protein forms with reduced anti-calcific activity and has been associated with accelerated vascular and soft-tissue calcification [6,8,13,25,26,27].

GRP, also known as upper zone of growth plate and cartilage matrix-associated protein (UCMA), has emerged as a particularly intriguing VKDP because of its exceptionally high density of Gla residues relative to its molecular size, which confers remarkable calcium-binding capacity [28,29,30]. The gene encoding GRP is officially designated *UCMA* according to the HUGO Gene Nomenclature Committee (HGNC); therefore, in this review, *UCMA* is used when referring to the gene, whereas GRP is used when referring to the protein [31]. Experimental data suggest that GRP may inhibit ectopic calcification directly by binding calcium–phosphate mineral phases and indirectly by participating in extracellular vesicles and calciprotein particles together with other calcification inhibitors such as MGP and fetuin-A [15,20,32,33]. In addition to its anti-calcific role, GRP has also been implicated in inflammatory modulation, including suppression of crystal-induced and macrophage-mediated inflammatory responses, suggesting that it may function at the interface of mineral homeostasis and immune signaling [30,34,35]. This broader functional profile is particularly relevant because calcification and inflammation are increasingly recognized as mutually reinforcing processes in chronic disorders such as CKD, calcific aortic valve disease, osteoarthritis, and tumor-associated microcalcification [34,36,37,38]. Although the available evidence supports an important role for GRP in pathological calcification and inflammation, several key questions remain unresolved, including the regulation of GRP expression, the functional differences between carboxylated and undercarboxylated forms, and the relative significance of circulating versus tissue-bound GRP pools [23,39,40,41]. The aim of this review is to provide a critical overview of current knowledge on GRP, with particular emphasis on its molecular characteristics, vitamin K dependency, anti-calcific and anti-inflammatory actions, and clinical relevance across cardiovascular, renal, skeletal, and other pathological settings. In addition, this review highlights current methodological limitations and knowledge gaps, including the lack of standardized assays capable of reliably distinguishing different GRP forms and the need for longitudinal studies to better define its biomarker and potential translational value [23,41,42].

## 2. *UCMA* Gene Expression and Its Regulation

The *UCMA* gene is evolutionarily conserved among vertebrates and is organized into five coding exons encoding a prepropeptide of approximately 135 amino acids. After removal of the signal peptide, proGRP undergoes further proteolytic processing by furin-like proteases, yielding a short propeptide and a mature GRP peptide of approximately 67–74 amino acids. The identification of UCMA orthologs across multiple vertebrate species, together with their apparent absence in invertebrates, supports the concept that GRP fulfills a conserved role in vertebrate tissue biology, particularly in tissues involved in regulated mineralization and extracellular matrix organization [28,29,30].

In addition to its basic gene structure, the genomic context of *UCMA* has been partially characterized. The *UCMA* gene is located on chromosome 10 and encodes multiple transcript variants, although the functional differences among these transcripts remain incompletely defined [31]. Genetic studies have identified several *UCMA* variants, including a common carboxy-terminal Thr138Ser polymorphism; however, its functional significance remains uncertain [43]. In addition, association studies have suggested a potential link between *UCMA* gene variants and skeletal disorders such as Paget’s disease of bone, although these findings remain limited and require further validation [44]. Overall, the clinical significance of *UCMA* genetic variation remains largely unexplored, particularly in cardiovascular and calcification-related diseases.

Expression studies have consistently shown that *UCMA*/GRP is highly enriched in cartilaginous tissues. During skeletal development, its expression spatially and temporally overlaps with established markers of chondrogenesis, particularly type II collagen (COL2), supporting a close association with the chondrocyte lineage. In vivo studies in mice and rats demonstrated that *UCMA* transcripts are present across several stages of chondrocyte differentiation, extending from proliferative to hypertrophic zones, which suggests that *UCMA*/GRP may participate throughout chondrocyte maturation rather than being restricted to a single differentiation stage [28,39,40,45,46].

**Figure 1:**
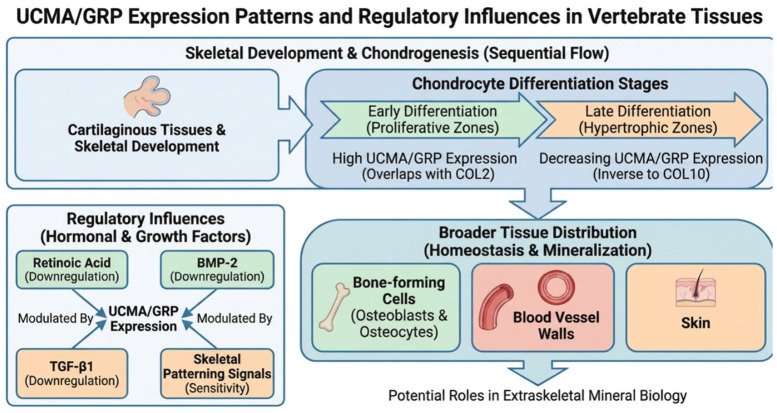
*UCMA*/GRP expression patterns and reported regulatory influences in vertebrate tissues. Schematic overview of the currently available evidence regarding *UCMA*/GRP expression during skeletal development and chondrocyte differentiation. *UCMA*/GRP expression is enriched in cartilaginous tissues and is highest during early differentiation stages, where it overlaps with type II collagen (COL2), and decreases during late hypertrophic differentiation, showing an inverse relationship with type X collagen (COL10). In addition to cartilage, *UCMA*/GRP expression has been reported in bone-forming cells, blood vessel walls, and skin, supporting a broader role in skeletal and extraskeletal mineral biology. The figure also summarizes the main reported regulatory influences on *UCMA*/GRP expression, including downregulation by retinoic acid, BMP-2, and TGF-β1, as well as sensitivity to skeletal patterning signals. Overall, the figure reflects a relatively consistent descriptive expression pattern, while highlighting that the upstream transcriptional regulation of *UCMA* remains incompletely defined.

Comparative analyses further indicate that *UCMA*/GRP displays a broader distribution across chondrocyte subpopulations than some other vitamin K-dependent proteins, such as matrix Gla protein (MGP), whose expression appears more restricted. This broader expression pattern supports the view that GRP may have functions beyond a narrow developmental window and may contribute more generally to cartilage homeostasis and matrix-related regulation [28,39]. Importantly, GRP expression is not confined to cartilage. Transcripts and/or protein expression have also been reported in bone-forming cells, including osteoblasts and osteocytes, as well as in extraskeletal tissues such as blood vessel walls and skin. These observations suggest that GRP is not merely a cartilage-specific developmental marker, but rather a more widely distributed regulator potentially involved in both skeletal and extraskeletal mineral biology [28,40,47,48]. In vitro studies using chondrogenic models have shown that GRP expression is highest during early stages of differentiation and gradually declines as cells progress toward hypertrophic maturation. GRP expression closely parallels COL2 expression and is inversely associated with type X collagen (COL10), a marker of chondrocyte hypertrophy. On this basis, GRP has been proposed as a useful marker of chondrocyte differentiation, and this application has been adopted in several developmental and cartilage-related experimental models [39,40,49]. The currently available evidence on *UCMA*/GRP expression patterns during chondrocyte differentiation, its broader tissue distribution, and the main reported regulatory influences is summarized schematically in Figure 1.

Despite these relatively consistent descriptive expression data, the upstream regulation of *UCMA* transcription remains insufficiently characterized. *UCMA* was originally identified as a chondrocyte-associated gene whose expression decreases during retinoic acid-induced dedifferentiation, suggesting that its transcription is linked to maintenance of the differentiated chondrocyte phenotype. In addition, *UCMA*/GRP expression has been reported to be downregulated in response to bone morphogenetic protein-2 (BMP-2) and transforming growth factor-β1 (TGF-β1), indicating that it may be sensitive to signaling pathways involved in skeletal patterning, matrix remodeling, and differentiation control [39,40].

Other experimental observations suggest that *UCMA*/GRP expression during limb development may occur independently of Indian hedgehog signaling, implying that *UCMA*/GRP is not simply integrated into the canonical hypertrophic chondrocyte regulatory axis [50]. In parallel, in silico promoter analyses have predicted potential binding sites for several transcription factors, including ETS family members, MEF2, E47, and STAT1. However, direct functional validation of these transcription factors in *UCMA* gene regulation is still lacking, and the currently available evidence remains largely descriptive or predictive rather than mechanistically definitive [29,39].

Taken together, the available evidence indicates that *UCMA*/GRP expression is tightly linked to skeletal development, chondrocyte differentiation, and mineralization-related tissues, while *UCMA* transcriptional regulation remains poorly understood. At present, the literature provides a reasonably coherent map of where and when *UCMA*/GRP is expressed, but only limited insight into the signaling pathways and transcriptional networks that govern *UCMA* expression under physiological and pathological conditions. This represents an important knowledge gap, particularly in view of the growing interest in GRP as a regulator of calcification and inflammation. Future studies should therefore focus on defining the upstream molecular drivers of *UCMA* expression, clarifying tissue-specific regulation, and determining how developmental, inflammatory, and calcification-related stimuli influence *UCMA* transcription in different biological contexts [23,29,30].

## 3. Vitamin K Dependency and γ-Carboxylation Status

Vitamin K (VK) is an essential cofactor for γ-glutamyl carboxylase, the enzyme responsible for the post-translational conversion of specific glutamate residues into γ-carboxyglutamate (Gla), a modification required for the biological activity of vitamin K-dependent proteins (VKDPs) [8,23,24,25]. Although this process is classically recognized for its role in the activation of hepatic coagulation factors, the identification of Gla-containing proteins in extrahepatic tissues established a broader family of VKDPs involved in bone biology, vascular homeostasis, mineralization control, and tissue remodeling [7,8,23,51,52,53].

Within this family, GRP is particularly distinctive because it represents the most densely γ-carboxylated protein identified to date. GRP is a small, secreted protein with an estimated molecular mass of approximately 10.2 kDa and contains an exceptionally high number of Gla residues relative to its molecular size [28,29,30]. GRP isolated from Adriatic sturgeon cartilage was shown to contain 16 Gla residues within a 74-amino acid mature sequence, conferring extraordinary calcium-binding capacity. Although the original biochemical characterization was performed in Adriatic sturgeon cartilage, a high density of predicted Gla residues is conserved across vertebrate GRP orthologs, including mammalian and human GRP. This supports the concept that dense γ-carboxylation is a central structure–function property of GRP rather than a species-specific feature [28,29]. This unusually high density of Gla residues is believed to underlie the strong mineral-binding properties of GRP and its proposed role as a regulator of extracellular calcium handling and pathological mineral deposition [22,28,29].

As with other VKDPs, the biological activity of GRP critically depends on its γ-carboxylation status. Carboxylated GRP (cGRP) is generally regarded as the functionally active form in the context of calcification inhibition, whereas undercarboxylated or non-carboxylated GRP (ucGRP) has markedly reduced anti-calcific capacity [20,30,34,41].

This distinction is of particular pathophysiological importance because accumulation of ucGRP has been associated with several calcification-related conditions, including calcific aortic valve disease, osteoarthritis, and tumor-associated microcalcifications. In these settings, ucGRP frequently predominates at sites of pathological mineral deposition, suggesting that impaired vitamin K-dependent activation may contribute to defective local inhibition of calcification [20,34,38,41]. Experimental and translational data further support a broader connection between vitamin K status and pathological calcification. Pharmacological inhibition of extrahepatic γ-carboxylation by warfarin induces medial vascular calcification in animal models, while higher vitamin K intake and restoration of vitamin K availability have been associated with reduced vascular calcification and improved vascular elasticity. In experimental settings, vitamin K supplementation has also been shown to attenuate or partially reverse pre-existing calcification [6,13,54,55,56,57,58].

In functional studies, both cGRP and ucGRP retain some degree of mineral-binding affinity; however, only cGRP has been shown to effectively inhibit calcification in vascular and articular tissues. These findings indicate that mineral binding alone is not sufficient to explain the full biological activity of GRP and that adequate γ-carboxylation is required for optimal anti-calcific function [20,34,41]. Interestingly, the role of γ-carboxylation appears to be less straightforward in inflammation. Emerging evidence suggests that the anti-inflammatory effects of GRP may be at least partially preserved even in the undercarboxylated form. Both cGRP and ucGRP have been reported to reduce the production of pro-inflammatory mediators such as tumor necrosis factor-α (TNF-α) and prostaglandin E2 (PGE-2) in immune and cartilage-related cell models [30,34,35]. These observations raise the possibility that the anti-calcific and anti-inflammatory functions of GRP are not equally dependent on γ-carboxylation. Whereas γ-carboxylation appears essential for efficient inhibition of mineral deposition, inflammatory modulation may involve additional structural domains or signaling interactions that are less strictly dependent on full Gla modification [23,34,35].

Nevertheless, the precise relationship between vitamin K availability, GRP carboxylation status, tissue localization, and biological activity remains incompletely resolved. In particular, it is still unclear to what extent circulating cGRP and ucGRP reflect tissue-level activity, whether partial carboxylation states have distinct functions, and how vitamin K status modifies GRP-mediated effects across different disease settings [23,41,42].

Current analytical approaches for distinguishing different GRP forms remain mainly research-based. Mass spectrometry-based proteomic methods can identify GRP in EVs, CPPs, mineral-bound protein fractions, and complex biological samples, whereas monoclonal antibody-based approaches have enabled experimental discrimination between carboxylated and undercarboxylated GRP forms in tissue and circulating samples. However, these approaches are not yet standardized, widely available, or validated for routine clinical use, and differences in assay design may partly explain variability across studies [15,20,42,47,59].

**Figure 2:**
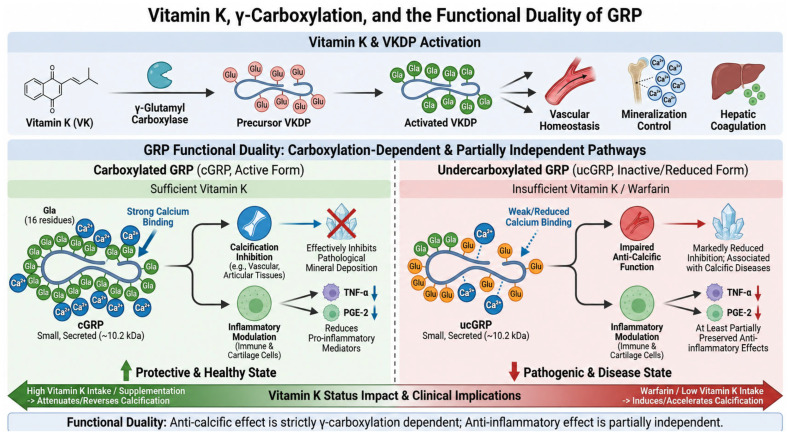
Vitamin K, γ-carboxylation, and the functional duality of GRP. Schematic representation of the role of vitamin K in γ-glutamyl carboxylase-mediated activation of vitamin K-dependent proteins and the functional consequences of GRP carboxylation status. Under conditions of sufficient vitamin K availability, carboxylated GRP (cGRP) displays strong calcium-binding capacity and effectively inhibits pathological mineral deposition, while also exerting anti-inflammatory effects in immune and cartilage-related cells. In contrast, under conditions of insufficient vitamin K availability or warfarin exposure, undercarboxylated GRP (ucGRP) exhibits reduced anti-calcific activity and impaired inhibition of pathological calcification, although anti-inflammatory effects may be at least partly preserved. Overall, the figure illustrates that the anti-calcific function of GRP is strongly γ-carboxylation-dependent, whereas its anti-inflammatory activity appears only partially dependent on full γ-carboxylation.

Collectively, the available evidence identifies γ-carboxylation as a central determinant of GRP anti-calcific function, while suggesting a more complex and potentially partially carboxylation-independent role in inflammatory regulation. This functional duality reinforces the importance of vitamin K status in modulating GRP biology and supports further investigation of GRP as both a biomarker of disturbed mineral homeostasis and a potential mechanistic contributor to calcification-associated disease [6,20,23,35]. A schematic overview of the relationship between vitamin K-dependent γ-carboxylation and the functional duality of GRP is shown in Figure 2.

## 4. Inhibition of Calcification: Direct and Indirect Mechanisms

The exceptionally high density of Gla residues within GRP gives this protein a unique biochemical profile among vitamin K-dependent proteins and supports its proposed role as a potent inhibitor of pathological calcification. Because GRP contains up to 16 Gla residues within a relatively short mature peptide, it displays the highest Gla-to-size ratio described to date, a feature that confers extraordinary calcium-binding potential [28,29,30].

Early biochemical studies demonstrated that GRP can be selectively extracted during demineralization of sturgeon extracellular matrix and that its Gla-rich domain is capable of binding calcium-containing mineral phases. These findings provided the first direct evidence that GRP may interact physically with calcium crystals and modulate mineral deposition at the extracellular level [28,29]. More broadly, the calcium-binding properties of Gla residues enable interaction not only with free calcium ions, but also with mineral phases such as hydroxyapatite. Similar principles apply to other vitamin K-dependent proteins involved in mineralization control, including matrix Gla protein (MGP) and osteocalcin (OC), although GRP appears particularly adapted to this function because of its unusually dense Gla content [7,8,22,23].

Functional studies in vascular and connective tissue models further support a direct anti-calcific effect of GRP. Supplementation with cGRP results in a clear, dose-dependent inhibition of calcification, whereas ucGRP shows only limited inhibitory activity. These observations indicate that γ-carboxylation is essential not merely for mineral binding, but for the full anti-calcific function of GRP [20,34,41].

However, the anti-calcific activity of GRP cannot be explained solely by direct crystal binding. Increasing evidence indicates that GRP also acts indirectly through the regulation of extracellular particles involved in mineral handling, particularly extracellular vesicles (EVs) and calciprotein particles (CPPs) [15,20,30].

Viegas and colleagues demonstrated that EVs released from healthy vascular smooth muscle cells (VSMCs) are enriched in GRP, MGP, and fetuin-A, whereas EVs generated under calcifying conditions contain reduced levels of GRP and MGP together with increased calcium content. This shift in EV composition suggests that GRP contributes to maintaining the anti-calcific phenotype of VSMC-derived EVs under physiological conditions and that loss of GRP enrichment may favor transition toward a pro-calcific vesicular phenotype [15,20]. This mechanism is biologically plausible because vascular calcification shares important features with physiological skeletal mineralization, including extracellular vesicle-mediated mineral nucleation and phenotypic transdifferentiation of VSMCs toward an osteoblast-like state. Within such calcification-prone microenvironments, GRP appears to function as part of a broader inhibitory network rather than as an isolated effector [2,3,18,20,60]. Indeed, GRP has been shown to form multiprotein complexes with other major calcification inhibitors, particularly MGP and fetuin-A. These complexes localize to sites of mineral deposition and are thought to cooperatively inhibit crystal nucleation, growth, and maturation. The association is especially relevant because both MGP and fetuin-A are well-established regulators of ectopic calcification at tissue and circulating levels [8,20,32,33].

Although GRP and MGP are both vitamin K-dependent inhibitors of ectopic calcification, the currently available evidence suggests that their functions are more likely complementary and synergistic than simply redundant. MGP is a well-established local inhibitor of vascular mineralization, whereas GRP appears to contribute particularly through high-affinity mineral binding and incorporation into EVs, CPPs, and mineral-bound protein complexes. Their co-localization within calcified tissues and extracellular particles therefore supports the concept that GRP and MGP participate in a cooperative inhibitory network that limits crystal nucleation, growth, and maturation in the vascular media [15,20,33,61].

Under physiological conditions, EVs and CPPs are thought to remain in a relatively stable and less pathogenic state because they are loaded with mineralization inhibitors such as fetuin-A, MGP, and GRP. In contrast, under pathological conditions, reduced GRP content within EVs and circulating CPPs is associated with impaired mineral buffering capacity, enhanced inflammatory potential, and increased propensity to induce VSMC calcification [6,15,20,62,63]. This indirect regulatory role may be particularly relevant in chronic kidney disease, where disturbances in phosphate metabolism and systemic mineral stress favor maturation of CPPs and generation of pro-calcific extracellular particles. In this setting, GRP deficiency or reduced GRP loading into EVs and CPPs may amplify both mineral deposition and inflammation [13,15,42,64,65].

Another important and unresolved aspect of GRP biology is the apparent discrepancy between tissue accumulation and circulating levels. Increased local GRP expression and protein deposition have been observed at sites of pathological calcification, whereas circulating GRP levels may decline or show inverse associations with calcification burden in some clinical settings [20,42,47,66]. One possible explanation is that GRP undergoes redistribution or sequestration at sites of mineral deposition, thereby reducing its systemic availability while increasing its local concentration within calcified tissues. Although this interpretation is biologically plausible, current evidence remains insufficient to determine whether circulating GRP accurately reflects tissue-level anti-calcific activity or instead represents a broader marker of disturbed mineral homeostasis [20,23,42].

Taken together, the available data support a dual anti-calcific role for GRP. First, GRP directly binds calcium-containing mineral phases and inhibits crystal formation in a γ-carboxylation-dependent manner. Second, GRP indirectly regulates extracellular mineral handling by stabilizing EVs and CPPs and by cooperating with other calcification inhibitors within multiprotein complexes. This multifaceted mode of action positions GRP as an important modulator of vascular mineral homeostasis and provides a mechanistic basis for its emerging relevance in calcification-associated disorders [15,20,29,30].

## 5. GRP in Inflammation and Immune Modulation

Calcification and inflammation are increasingly recognized as closely interconnected processes that frequently coexist in chronic diseases such as atherosclerosis, chronic kidney disease, and osteoarthritis, where affected tissues are characterized by infiltration of monocytes, accumulation of macrophages, and deposition of calcium-containing mineral phases [3,34,36,37,67].

Within this context, GRP has emerged as a potentially important mediator linking mineral deposition to immune activation. Viegas and colleagues demonstrated that both GRP and matrix Gla protein (MGP) are expressed and translated by human leukocyte subsets and may be released into peripheral tissues or the circulation as components of extracellular vesicles (EVs). These observations broaden the biological scope of GRP beyond mineralized connective tissues and support a role in immune cell biology [23,35]. Experimental studies further suggest that GRP may act as both an endogenous and exogenous anti-inflammatory factor in monocytic THP-1 cells and THP-1–derived macrophages. Importantly, although γ-carboxylation appears to occur in immune cells, the currently available evidence indicates that γ-carboxylation is not the principal determinant of GRP anti-inflammatory activity, in contrast to its clearly established importance for calcification inhibition [30,34,35]. This distinction is mechanistically important because it suggests that the anti-calcific and anti-inflammatory functions of GRP may not rely equally on the same structural requirements. Whereas the inhibition of mineral deposition is strongly dependent on adequate γ-carboxylation, inflammatory modulation may involve additional protein interactions or signaling effects that are at least partly retained in undercarboxylated GRP [23,34,35].

In osteoarthritis, GRP has been shown to exert anti-inflammatory effects in synoviocytes and chondrocytes, further supporting a broader immunomodulatory role in tissues affected by chronic low-grade inflammation and pathological calcification. However, despite these observations, the precise contribution of GRP to immune cell-driven inflammatory responses remains incompletely defined [34,41,68,69].

Mechanistically, GRP has been reported to attenuate monocyte and macrophage pro-inflammatory responses by reducing the production of key mediators such as interleukin-1β (IL-1β), tumor necrosis factor-α (TNF-α), prostaglandin E2 (PGE-2), and nuclear factor-κB (NF-κB)-related inflammatory signaling. This is particularly relevant because these pathways are central to the amplification of chronic inflammation and are also implicated in osteogenic conversion and vascular injury [34,35,36]. Among these mediators, TNF-α is of particular interest because macrophage-derived TNF-α promotes atherosclerosis, vascular calcification, endothelial dysfunction, and osteogenic differentiation of vascular smooth muscle cells (VSMCs), thereby linking immune activation directly to vascular remodeling and atherogenesis [70]. Experimental inhibition of TNF-α signaling in ApoE-deficient mice has been shown to reduce aortic lesion size, underscoring the central role of this cytokine in vascular inflammatory disease [36,37,71].

Consistent with this framework, Viegas et al. demonstrated that GRP expression is upregulated in THP-1 monocytes and macrophages following inflammatory stimulation with hydroxyapatite crystals or lipopolysaccharide (LPS). Treatment of THP-1–derived macrophages with purified cGRP or ucGRP, as well as with basic calcium phosphate particles coated with GRP-containing protein–mineral complexes, resulted in reduced production of TNF-α and PGE-2. In addition, GRP overexpression counteracted inflammatory responses induced by hydroxyapatite and LPS, supporting a direct anti-inflammatory role for GRP in crystal- and pathogen-associated inflammatory conditions [34,35]. These findings are especially relevant because calcium-containing crystals are not biologically inert, but can themselves function as inflammatory stimuli that activate innate immune responses [72]. GRP may therefore attenuate a self-amplifying loop in which mineral deposition promotes macrophage activation, inflammatory cytokine release, and further calcification [34,35,37].

**Figure 3:**
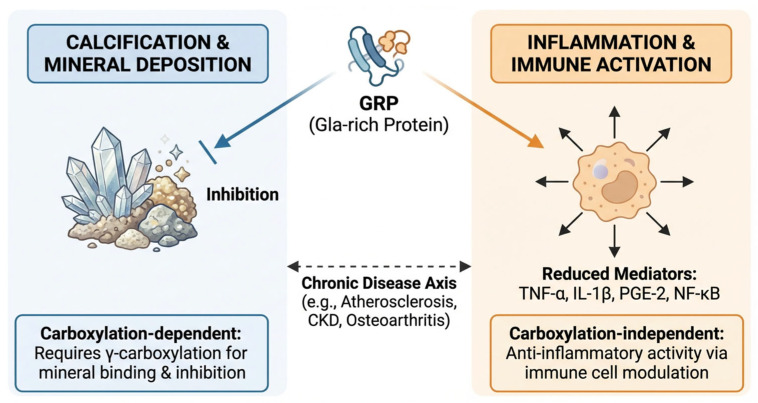
Proposed dual role of Gla-rich protein (GRP) at the interface of calcification and inflammation. Schematic representation of the two major functional axes through which GRP may act in chronic calcification-associated disorders. On the left, GRP inhibits calcification and mineral deposition through a mechanism that depends on adequate γ-carboxylation, which is required for efficient mineral binding and anti-calcific activity. On the right, GRP attenuates inflammation and immune activation by reducing pro-inflammatory mediators, including TNF-α, IL-1β, PGE-2, and NF-κB-related signaling. In contrast to its anti-calcific role, the anti-inflammatory effects of GRP appear to be at least partly independent of full γ-carboxylation. Together, these actions position GRP as a mechanistic link within the calcification–inflammation axis in chronic diseases such as atherosclerosis, chronic kidney disease, and osteoarthritis.

Taken together, the available evidence supports the view that GRP acts as an important modulator of immune cell-mediated inflammation in chronic inflammatory and calcification-associated disorders. Rather than functioning solely as a mineral-binding protein, GRP appears to operate at the interface between extracellular mineral deposition and innate immune signaling, thereby serving as a potential molecular link within the inflammation–calcification axis [23,30,34,35]. The proposed dual role of GRP at the intersection of calcification and inflammation is summarized in Figure 3.

## 6. *UCMA*/GRP and Osteogenesis

UCMA, also known as GRP, is a secreted protein expressed in fetal and juvenile growth plate cartilage as well as in trabecular bone, indicating that its biological role extends beyond vascular and inflammatory contexts into skeletal development and bone-related differentiation processes [28,29,40]. During long bone development, *UCMA*/GRP expression has been detected in resting chondrocytes, osteocytes, and osteoblasts within trabecular bone, supporting a role in both chondrogenic and osteogenic compartments of the developing skeleton [39,40].

Early experimental work suggested that *UCMA*/GRP may act as a negative regulator of osteogenic differentiation. Surmann-Schmitt and colleagues reported that *UCMA* is expressed at early stages of osteoblast differentiation and that recombinant *UCMA* interferes with osteoblast maturation, implying that *UCMA*/GRP may restrain premature progression toward a fully mature osteoblastic phenotype [40]. This concept is consistent with findings in non-skeletal calcification models, where GRP has also been proposed as a negative regulator of osteogenic conversion [73,74]. In vascular smooth muscle cells (VSMCs), carboxylated GRP (cGRP) has been shown to inhibit calcification and osteochondrogenic differentiation by upregulating α-smooth muscle actin (α-SMA) and downregulating osteopontin (OPN), thereby helping preserve a more contractile, non-osteogenic phenotype [2,3,20]. However, other studies support a more complex and potentially pro-osteogenic role for *UCMA*/GRP under specific developmental or differentiation conditions. GRP expression has been reported to be upregulated by the osteogenic transcription factors Runx2 and osterix (Osx), and this upregulation has been associated with enhanced osteoblast differentiation and increased mineralized nodule formation [40,75]. Conversely, GRP expression has also been shown to be downregulated by BMP-2 in chondrogenic cells, indicating that its regulation is not linear and may depend strongly on cellular lineage, developmental timing, and the local signaling milieu [39,40,75].

Taken together, these observations suggest that GRP regulation during osteogenesis is highly context-dependent and may differ according to whether the relevant biological setting is physiological bone development, chondrocyte maturation, or ectopic osteogenic transdifferentiation in pathological tissues [20,30,40]. This broader context is important because blood vessels exposed to calcifying conditions can activate a transcriptional program that closely resembles osteogenesis. Under such conditions, vascular tissues express multiple osteogenic transcription factors, including Msx2, Sox9, Runx2, and Osx, together with bone-associated proteins such as BMP-2, osteopontin, osteonectin, type I collagen, bone sialoprotein, osteocalcin, MGP, and fetuin-A [1,2,18].

Supporting a role in osteoblast maturation rather than simple inhibition, Lee and colleagues reported enhanced mineralized nodule formation in *UCMA*-overexpressing osteoblasts and in cells cultured in *UCMA*-containing medium. *UCMA* overexpression was associated with increased expression of osteocalcin, a marker of late-stage osteoblast differentiation, as well as elevated OPN levels, suggesting a shift toward a more mature osteoblastic phenotype [75]. In vivo evidence regarding the role of *UCMA*/GRP in skeletal development remains conflicting. Neacsu et al. reported that *UCMA* knockdown in zebrafish leads to impaired skeletal development and severe growth retardation, suggesting an important role in skeletogenesis [76]. In contrast, Eitzinger et al. showed that *UCMA*-deficient mice exhibit normal skeletal development, indicating that *UCMA* is not essential for endochondral ossification in mammals [77]. These apparently divergent findings may reflect species-specific biology, developmental compensation, redundancy with other matrix-associated proteins, or differences between early developmental patterning and postnatal skeletal maintenance [40,74,76,77].

**Figure 4:**
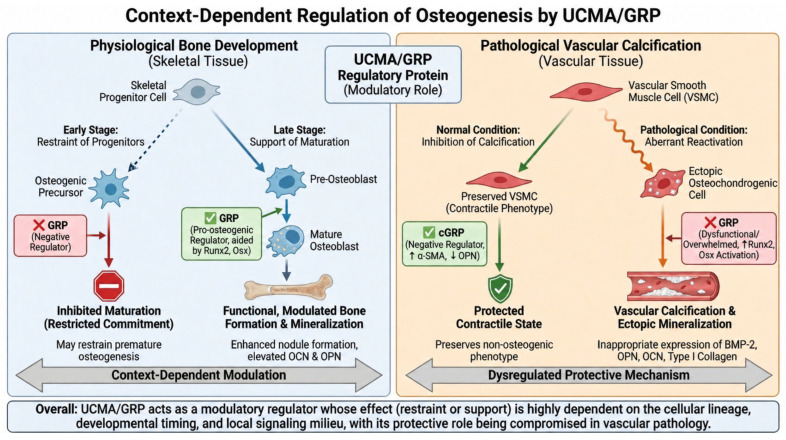
Context-dependent regulation of osteogenesis by *UCMA*/GRP. Schematic representation of the proposed context-dependent role of *UCMA*/GRP in physiological skeletal development and pathological vascular calcification. In skeletal tissue, *UCMA*/GRP may exert stage-dependent effects, including restraint of early osteogenic commitment and support of later osteoblast maturation and mineralized nodule formation under specific developmental conditions. In vascular tissue, carboxylated GRP (cGRP) is proposed to preserve the contractile phenotype of vascular smooth muscle cells and inhibit osteochondrogenic transdifferentiation and ectopic mineralization. Under pathological calcifying conditions, dysfunction or loss of this protective GRP-associated regulation may contribute to vascular calcification and aberrant activation of osteogenic programs. Overall, *UCMA*/GRP is depicted as a modulatory rather than indispensable regulator whose effects depend on cellular lineage, developmental timing, and local signaling context.

Collectively, the available evidence indicates that *UCMA*/GRP acts as a modulatory rather than indispensable regulator of osteogenesis. Depending on the biological context, it may either restrain early osteogenic commitment or support later stages of osteoblast maturation and matrix mineralization. This duality is particularly relevant for understanding GRP not only in physiological skeletal biology, but also in pathological settings such as vascular calcification, where osteogenic programs are aberrantly reactivated [20,30,40,75]. The context-dependent effects of *UCMA*/GRP across physiological skeletal development and pathological vascular calcification are summarized schematically in Figure 4.

## 7. GRP and Associated Pathologies

### 7.1. Gla-Rich Protein and Chronic Kidney Disease

Cardiovascular disease (CVD) is the leading cause of morbidity and mortality in patients with chronic kidney disease (CKD), and this excess risk cannot be explained solely by traditional cardiovascular risk factors [78]. Beyond hypertension, diabetes, and dyslipidemia, CKD is characterized by profound disturbances in mineral metabolism and endocrine regulation, collectively encompassed by the concept of CKD–mineral and bone disorder (CKD-MBD) [3,9,10,13]. CKD-MBD is marked by abnormalities in phosphate and calcium balance, parathyroid hormone regulation, vitamin D metabolism, fibroblast growth factor-23 (FGF-23), and Klotho signaling, all of which contribute to vascular dysfunction and adverse cardiovascular outcomes [9,10,13,14,79].

Within this pathophysiological environment, vascular calcification emerges as a central non-traditional cardiovascular risk factor. Phosphate overload, elevated calcium–phosphate product, increased FGF-23, and reduced α-Klotho promote osteochondrogenic transdifferentiation of vascular smooth muscle cells (VSMCs), extracellular vesicle release, and progressive mineral deposition within the vascular wall [2,3,6,13,80].

Against this background, GRP has attracted increasing attention as a potential biomarker and mechanistic modulator of disturbed mineral homeostasis in CKD. Silva et al. investigated the relationship among circulating GRP levels, renal function, and vascular calcification and demonstrated a strong positive correlation between estimated glomerular filtration rate (eGFR) and serum GRP concentrations. Progressive decline in eGFR from CKD stage 2 to stage 4 was accompanied by a marked reduction in circulating GRP levels, suggesting that GRP may represent an early marker associated with deterioration of kidney function [42]. In that study, lower circulating GRP levels were also associated with markers of vascular calcification, altered mineral metabolism, and increased pulse pressure in adult diabetic patients with CKD, supporting a link between reduced GRP availability and increased cardiovascular risk [42]. These findings are biologically plausible because GRP is not only a circulating protein, but also a constituent of extracellular structures involved in mineral buffering.

Viegas et al. identified GRP as a constitutive component of circulating extracellular vesicles (EVs) and calciprotein particles (CPPs), both of which are highly relevant to the pathogenesis of vascular calcification in CKD [15,20]. In patients with advanced CKD, reduced GRP content within EVs and CPPs was associated with enhanced mineral maturation, increased inflammatory potential, and greater capacity to induce VSMC calcification. These data suggest that GRP deficiency within extracellular particles may impair mineral buffering and favor transition toward a more pathogenic, pro-calcific, and pro-inflammatory phenotype [15]. Importantly, supplementation of CPPs isolated from CKD patients with GRP attenuated calcification, inflammation, and osteogenic differentiation in VSMCs, providing functional support for a protective role of GRP in the CKD calcification milieu [15].

Taken together, these observations position GRP at the intersection of several key processes relevant to CKD-MBD, including mineral stress, extracellular particle maturation, vascular calcification, and inflammation. This supports the view that GRP may be more than a passive biomarker and could participate directly in the regulation of calcification-prone microenvironments in CKD [15,23,42]. Nevertheless, important uncertainties remain. It is still unclear whether reduced circulating GRP levels contribute causally to vascular calcification or primarily reflect disease severity, altered vitamin K-dependent activation, or redistribution of GRP into calcified tissues and extracellular particles. In addition, the clinical significance of total circulating GRP may differ from that of particle-bound or tissue-bound GRP, and current human data are largely cross-sectional [15,23,42].

Therefore, although currently available evidence strongly supports an association between reduced GRP activity and adverse calcification-related processes in CKD, further longitudinal and mechanistic studies are required to determine whether GRP has independent predictive value and whether it represents a modifiable target within the CKD-MBD spectrum [13,23,42].

### 7.2. Gla-Rich Protein and Calcific Aortic Valve Disease

Vascular calcification is a well-established predictor of coronary heart disease and also plays a central role in the pathogenesis of calcific aortic valve disease (CAVD), a progressive disorder that leads to aortic valve stenosis and substantial cardiovascular morbidity and mortality [81,82,83,84]. Similar to vascular calcification, CAVD is no longer regarded as a passive degenerative consequence of aging, but rather as an active and tightly regulated pathological process involving inflammation, extracellular matrix remodeling, osteogenic signaling, and progressive mineral deposition [20,85,86,87]. Despite these mechanistic parallels, data specifically addressing the role of GRP in CAVD remain limited. Nevertheless, the available evidence suggests that GRP may participate in the local regulation of valvular calcification in a manner analogous to its proposed role in vascular tissues [20,23,30].

Although both carboxylated GRP (cGRP) and undercarboxylated GRP (ucGRP) retain calcium/phosphate mineral-binding affinity and are detectable in healthy connective tissues, preferential accumulation of ucGRP has been associated with pathological calcification [20,38,41]. In calcified aortic valves, Viegas et al. demonstrated that both GRP forms are expressed in vascular smooth muscle cells and valvular interstitial cells (VICs) and accumulate at sites of mineral deposition, with ucGRP predominating in calcified regions. This predominance of ucGRP suggests that impaired vitamin K-dependent activation of GRP may contribute to defective local inhibition of crystal growth within diseased valves [20]. In contrast, cGRP appears to be selectively enriched within mineral-bound protein fractions, raising the possibility that limited amounts of functionally active GRP are recruited to calcification sites as part of a local compensatory anti-calcific response [20].

Proteomic analyses of mineral-bound protein extracts from calcified aortic valves have confirmed the presence of GRP together with other major inhibitors and modulators of pathological calcification, including matrix Gla protein (MGP) and fetuin-A, as well as additional proteins involved in vascular and skeletal mineralization [8,20,32]. These findings support the concept that CAVD shares important molecular features with vascular calcification and physiological biomineralization, including extracellular matrix remodeling, osteogenic differentiation of resident cells, and local assembly of multiprotein mineral-binding complexes [88]. Within this framework, GRP appears to function not as an isolated factor, but as part of a broader inhibitory network that may modulate nucleation, growth, and maturation of pathological mineral deposits in the aortic valve [20,23,30,82,83].

However, the currently available evidence remains largely descriptive, and several important questions remain unresolved. In particular, it is still unclear whether altered GRP carboxylation plays a causal role in CAVD progression, whether circulating GRP reflects valvular calcification activity, and whether vitamin K-dependent modulation of GRP has clinical relevance in this setting [20,23,89,90,91].

Overall, the limited available data suggest that GRP is involved in the local biology of calcified aortic valves, particularly through differential distribution of cGRP and ucGRP at sites of mineral deposition. This supports the view that GRP may represent one component of the regulatory machinery governing pathological valvular calcification, although its precise mechanistic and clinical significance in CAVD remains to be established [20,23,30].

### 7.3. Gla-Rich Protein in Osteoarthritis

Osteoarthritis (OA) is a degenerative joint disease characterized by chronic low-grade inflammation, synovial activation, abnormal bone remodeling, and progressive loss of articular cartilage. Although traditionally regarded as a primarily “wear-and-tear” disorder, OA is now understood as a biologically active disease involving complex interactions among inflammation, matrix degradation, chondrocyte dysfunction, and ectopic mineralization [92,93,94,95].

A growing body of evidence indicates that pathological mineralization contributes to OA progression. Deposition of basic calcium phosphate crystals within the extracellular matrix promotes local inflammation and tissue damage, and crystal deposition has been reported not only in articular cartilage, but also in the synovial membrane and synovial fluid, where it may further amplify inflammatory responses [34,41,96,97]. Within this framework, vitamin K-dependent proteins (VKDPs) have attracted interest as potential modulators of OA-related calcification and inflammation. Vitamin K itself has been proposed to exert protective effects in joint tissues, at least in part through post-translational activation of extrahepatic VKDPs. Among these proteins, osteocalcin (OC), matrix Gla protein (MGP), and Gla-rich protein (GRP) have all been implicated in osteoarthritic tissues [8,23,68].

GRP appears particularly relevant because it combines two functions that are both central to OA pathobiology: inhibition of extracellular matrix calcification and modulation of inflammatory signaling. Although γ-carboxylation is essential for the anti-calcific activity of GRP, both carboxylated (cGRP) and undercarboxylated (ucGRP) forms have been detected in osteoarthritic cartilage and synovial tissue, with ucGRP predominating at sites of ectopic mineralization [34,41]. Cavaco et al. demonstrated that GRP gene expression is upregulated in OA-derived synoviocytes and chondrocytes and is associated with increased expression of calcification inhibitors such as MGP, as well as OA-related markers including cartilage oligomeric matrix protein, osteocalcin, and type X collagen. These findings support the concept that GRP is integrated into the broader molecular response of joint tissues to calcification stress and matrix remodeling [34]. In the same study, GRP progressively reduced the expression of inflammatory mediators such as cyclooxygenase-2 (COX-2) and matrix metalloproteinase-13 (MMP-13) in OA-derived synoviocyte and chondrocyte cultures. Moreover, treatment with basic calcium phosphate crystals coated with GRP attenuated crystal-induced inflammatory responses, and this effect appeared to occur independently of γ-carboxylation status [34]. These observations are particularly important because they suggest that GRP may directly modulate OA-associated inflammation in addition to limiting calcification. In other words, GRP may attenuate the self-reinforcing cycle in which crystal deposition promotes inflammation and inflammation, in turn, facilitates further tissue damage and pathological mineralization [34,68,95].

Additional support for a protective role of *UCMA*/GRP in joint tissues comes from experimental studies by Stock et al., who showed that *UCMA* is overexpressed in human and murine osteoarthritic cartilage compared with healthy controls. *UCMA*-deficient mice exhibited increased susceptibility to OA-associated cartilage damage and chondrocyte death, accompanied by enhanced subchondral bone turnover and osteoclastogenesis [98]. Importantly, *UCMA* was also shown to protect cartilage from aggrecan degradation by inhibiting ADAMTS-dependent aggrecanase activity, providing a plausible mechanism by which *UCMA*/GRP may preserve cartilage integrity in osteoarthritis. This finding extends the role of *UCMA*/GRP beyond mineral handling and inflammation toward direct regulation of extracellular matrix preservation [98,99].

Taken together, the available evidence indicates that *UCMA*/GRP acts as a multifunctional protective factor in osteoarthritis. By modulating ectopic mineralization, attenuating inflammatory signaling, and limiting matrix degradation, GRP appears to operate at the intersection of several key pathological pathways in OA. Nevertheless, the currently available evidence remains relatively limited, and further studies are needed to clarify whether GRP has independent biomarker value or therapeutic relevance in joint disease [34,41,68,98].

### 7.4. Gla-Rich Protein in Carcinoma

A growing body of evidence indicates that vitamin K (VK) and vitamin K-dependent proteins (VKDPs) participate in cancer biology, although their effects appear to be highly context-dependent and may vary according to tumor type, tissue microenvironment, and disease stage [100,101]. Proteins such as Gas6, matrix Gla protein (MGP), and osteocalcin (OC) have all been implicated in processes relevant to tumor progression, including proliferation, survival signaling, extracellular matrix remodeling, and pathological mineralization [30,38]. Within this broader framework, the identification of GRP as an additional VK-dependent protein associated with tumor-associated calcification is of particular interest [102]. Although the role of GRP in carcinogenesis itself remains poorly defined, its known involvement in mineral binding, calcification inhibition, and tissue remodeling makes it a plausible candidate for participation in pathological processes occurring in calcifying tumor microenvironments [23,30,38].

Viegas et al. investigated the distribution of GRP in human breast and skin carcinomas and demonstrated distinct accumulation patterns of carboxylated GRP (cGRP) and undercarboxylated GRP (ucGRP) in healthy and tumorous tissues. In normal skin and mammary gland tissues, both GRP forms were found to co-localize, suggesting that partial or incomplete γ-carboxylation may occur under physiological conditions [38]. In contrast, tumor-associated microcalcifications were characterized by a predominance of ucGRP together with reduced cGRP staining. This pattern suggests that impaired γ-carboxylation of GRP may be associated with pathological mineralization in malignant tissues and raises the possibility that functionally active GRP is relatively deficient at sites of tumor calcification [38]. These findings are biologically relevant because microcalcifications are not merely passive histological bystanders, but important features of the tumor microenvironment, particularly in breast carcinoma, where they may reflect altered mineral handling, extracellular matrix remodeling, and local tissue degeneration [101,103]. In this setting, altered GRP carboxylation may represent one component of a broader disturbance in VK-dependent regulation of pathological biomineralization [30,38].

At present, however, the available evidence does not support a direct causal role of GRP in tumor initiation or progression. Rather, current data suggest that GRP is more likely to reflect altered mineral metabolism within the tumor microenvironment than to function as a validated driver of carcinogenesis [23,30,38]. Nevertheless, the observed predominance of ucGRP in tumor-associated microcalcifications is noteworthy because it extends the relevance of GRP beyond vascular, renal, and articular disease into malignant tissues. This observation also reinforces the broader concept that GRP may serve as a VK-responsive component of pathological calcification across multiple clinical settings [23,38].

Further studies are needed to clarify whether GRP has biomarker potential in calcifying tumors, whether its carboxylation status carries diagnostic or prognostic information, and whether altered GRP distribution in carcinoma is simply an epiphenomenon or part of a biologically relevant mineralization pathway within the tumor niche [30,38].

**Figure 5:**
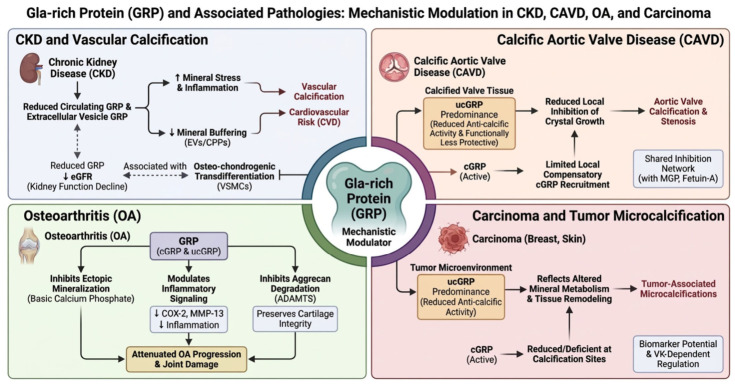
Gla-rich protein (GRP) and associated pathologies: mechanistic modulation in chronic kidney disease, calcific aortic valve disease, osteoarthritis, and carcinoma. Schematic summary of the proposed disease-specific roles of GRP. In chronic kidney disease, reduced circulating and extracellular vesicle-associated GRP is linked to impaired mineral buffering, vascular calcification, and cardiovascular risk. In calcific aortic valve disease, undercarboxylated GRP predominates in calcified valve tissue, whereas carboxylated GRP may represent a limited local compensatory anti-calcific response. In osteoarthritis, GRP is proposed to inhibit ectopic mineralization, attenuate inflammatory signaling, and limit cartilage degradation. In carcinoma-associated microcalcifications, altered distribution of carboxylated and undercarboxylated GRP suggests disturbed vitamin K-dependent regulation of pathological biomineralization. Overall, GRP is depicted as a context-dependent modulator linking mineral handling, inflammation, and tissue remodeling across multiple pathological settings.

The major disease-specific mechanisms through which GRP may modulate pathological calcification, inflammation, and tissue remodeling across chronic kidney disease, calcific aortic valve disease, osteoarthritis, and carcinoma are summarized in Figure 5.

An overview of the molecular mechanisms and clinical implications of GRP/*UCMA* across different disease settings is provided in Table 1.

### 7.5. Integrative Mechanistic Framework of GRP Across Disease States

Collectively, the available evidence indicates that GRP functions as a multifunctional modulator rather than a single-pathway effector, exerting context-dependent effects across vascular, renal, skeletal, articular, and malignant tissues. Despite the heterogeneity of these clinical settings, the biological role of GRP appears to converge around a limited number of interconnected mechanisms linking mineral handling, extracellular particle biology, and inflammatory regulation [20,23,30].

The first major pathway involves direct regulation of mineral deposition. Fully carboxylated GRP binds calcium–phosphate mineral phases through its γ-carboxyglutamate-rich domain and thereby inhibits hydroxyapatite nucleation, crystal growth, and extracellular matrix mineralization. This anti-calcific activity depends strongly on adequate γ-carboxylation and therefore on vitamin K availability, whereas undercarboxylated GRP exhibits reduced protective capacity and repeatedly predominates at sites of pathological calcification [20,28,29,34,41].

A second major pathway involves the regulation of extracellular particles, particularly calciprotein particles and extracellular vesicles. GRP contributes to maintaining these structures in a less crystalline and less inflammatory state, whereas reduced GRP content is associated with impaired mineral buffering capacity, enhanced pro-calcific potential, and increased capacity to induce vascular smooth muscle cell osteogenic differentiation. This mechanism appears especially relevant in chronic kidney disease, where systemic mineral stress promotes extracellular particle maturation and progressive vascular injury [15,20,23,42].

A third pathway involves modulation of inflammatory signaling. Experimental studies indicate that GRP attenuates crystal- and macrophage-associated inflammatory responses by reducing the production of mediators such as TNF-α, IL-1β, PGE-2, and NF-κB-related signaling. In contrast to its anti-calcific effects, these anti-inflammatory actions appear to be at least partly preserved independently of full γ-carboxylation, suggesting that the calcification-inhibitory and immunomodulatory functions of GRP are related but not mechanistically identical [23,30,34,35].

This integrated framework helps explain why altered GRP biology has been linked to several disease states. In chronic kidney disease, reduced circulating and particle-bound GRP is associated with vascular calcification, disturbed mineral metabolism, and cardiovascular risk. In calcific aortic valve disease, predominance of undercarboxylated GRP at sites of mineral deposition suggests impaired local inhibition of crystal growth. In osteoarthritis, GRP appears to modulate the interaction among calcification, inflammation, and matrix degradation, thereby contributing to cartilage protection. In tumor-associated microcalcifications, altered distribution of carboxylated and undercarboxylated GRP suggests that GRP may also participate in pathological biomineralization within malignant tissues [15,20,34,38,42,98].

Importantly, these observations do not support a uniform disease-specific role for GRP, but rather position it as a context-sensitive regulator whose biological effects depend on carboxylation status, tissue localization, extracellular binding partners, and the surrounding inflammatory and mineral milieu. This also helps explain why GRP may appear protective in some settings, compensatory in others, and incompletely effective at sites of advanced pathological calcification [20,23,30].

Taken together, the currently available data support a conceptual model in which GRP operates at the crossroads of three interconnected processes: mineral binding and crystal regulation, extracellular particle stabilization, and inflammation modulation. Disruption of any of these pathways may amplify ectopic calcification, chronic inflammation, and tissue remodeling. GRP should therefore be viewed as an integrative regulator of pathological biomineralization rather than as a disease-specific effector confined to a single organ system [15,20,23,30,34].

## 8. Clinical Implications and Future Directions

Accumulating experimental and clinical evidence indicates that GRP may have relevant clinical implications in diseases characterized by disturbed mineral metabolism, chronic inflammation, and pathological calcification. Among these, chronic kidney disease (CKD) currently represents the most clinically informative setting, because the association between GRP, vascular calcification, and mineral dysregulation has been most consistently explored in this population [15,23,42].

In patients with CKD, circulating GRP levels decline with worsening renal function and have been reported to correlate with estimated glomerular filtration rate, vascular calcification, pulse pressure, and markers of altered mineral metabolism. These findings suggest that GRP may serve as an early biomarker of vascular dysfunction and cardiovascular risk within the spectrum of CKD–mineral and bone disorder [42]. In line with these observations, more recent clinical data in patients undergoing peritoneal dialysis indicate that circulating total GRP levels are inversely associated with vascular calcification burden and are also related to inflammatory and mineral metabolism parameters, further supporting the potential biomarker relevance of GRP in advanced kidney disease [23,105].

The clinical relevance of GRP may depend not only on total circulating concentration, but also on its localization and functional status [106]. GRP has been identified as a constitutive component of calciprotein particles and extracellular vesicles, and reduction in GRP content within these structures is associated with enhanced pro-calcific and pro-inflammatory activity, particularly in advanced CKD [15,20].

Emerging evidence further suggests that the biological and potentially clinical significance of GRP depends on its γ-carboxylation status. Preferential accumulation of undercarboxylated GRP has been observed at sites of pathological calcification in vascular tissue, calcified aortic valves, osteoarthritic cartilage, and tumor-associated microcalcifications, whereas carboxylated GRP appears to be selectively enriched in mineral-bound protein fractions, possibly reflecting a local compensatory anti-calcific response [20,38,41].

These observations support the concept that altered vitamin K-dependent activation of GRP may contribute to impaired regulation of ectopic calcification across multiple chronic diseases [104]. From a translational perspective, this raises interest in vitamin K status as a potentially modifiable determinant of GRP activity [6,8,23,25].

However, despite this biological plausibility, current evidence does not justify GRP-targeted clinical intervention. Experimental studies support the importance of vitamin K-dependent γ-carboxylation in limiting pathological calcification, but human data remain insufficient to demonstrate that direct modulation of GRP translates into improved clinical outcomes. At present, GRP should therefore be regarded primarily as a biomarker candidate reflecting disturbed mineral homeostasis rather than as a validated therapeutic target [6,23,55,57,61,90].

Several important limitations currently restrict the immediate clinical translation of GRP. First, most available human studies are cross-sectional and therefore cannot determine causality or establish predictive value. Second, standardized assays capable of reliably distinguishing circulating carboxylated and undercarboxylated GRP are still lacking [59]. Third, the relative importance of circulating, particle-bound, and tissue-bound GRP pools remains insufficiently understood [15,23,41,42].

Additional uncertainty arises from the context-dependent biology of GRP. Its anti-calcific function appears strongly dependent on γ-carboxylation, whereas its anti-inflammatory effects may be at least partly preserved independently of full carboxylation. This functional complexity complicates both biomarker interpretation and therapeutic translation [23,30,34,35].

Future research should therefore focus on several priorities. These include assay standardization, clarification of the relative contributions of total versus carboxylated and undercarboxylated GRP, better characterization of circulating versus tissue-bound GRP pools, and longitudinal studies evaluating whether GRP independently predicts cardiovascular and calcification-related outcomes [15,23,42].

Particular emphasis should be placed on CKD and other calcification-prone disorders, where GRP biology appears most clinically relevant. In parallel, mechanistic studies are needed to determine whether altered GRP distribution merely reflects disease activity or actively contributes to the progression of calcification, inflammation, and tissue remodeling [15,20,34,38,42].

Taken together, the currently available evidence supports GRP as a promising integrative biomarker candidate at the intersection of mineral metabolism, inflammation, and pathological calcification. Nevertheless, substantial methodological and translational gaps remain, and further work is required before GRP can be incorporated into routine risk stratification or considered a therapeutic target in clinical practice [13,23,30].

## 9. Literature Search Strategy and Methodological Approach

A structured literature search was conducted to identify relevant studies on Gla-rich protein (GRP/*UCMA*), vitamin K-dependent proteins, ectopic calcification, inflammation, and mineral homeostasis. Searches were performed in PubMed, Scopus, and Web of Science for articles published up to April 2026, using combinations of controlled vocabulary terms, where available, and free-text keywords. Search terms included: “Gla-rich protein”, “GRP”, “*UCMA*”, “upper zone of growth plate and cartilage matrix-associated protein”, “vitamin K-dependent proteins”, “gamma-carboxylation”, “vascular calcification”, “chronic kidney disease”, “calciprotein particles”, “extracellular vesicles”, “inflammation”, “calcific aortic valve disease”, “osteoarthritis”, and “tumor microcalcifications”. In addition, reference lists of relevant articles were manually screened to identify further eligible studies.

Studies were screened based on titles and abstracts, followed by full-text evaluation where available. Eligible publications included original experimental studies, clinical observational studies, translational studies, and relevant reviews addressing GRP/*UCMA* biology, regulation, vitamin K dependency, calcification inhibition, immune modulation, and disease associations. Non-English publications, conference abstracts, and studies not directly relevant to GRP/*UCMA* or related vitamin K-dependent calcification pathways were excluded. For a limited number of older or otherwise difficult-to-access publications, information available from abstracts and bibliographic records was also considered when directly relevant to specific points of discussion.

This review was designed as a narrative synthesis integrating heterogeneous evidence across molecular, experimental, translational, and clinical domains. Because of substantial heterogeneity in study designs, biological models, measured GRP forms, clinical populations, and reported outcomes, quantitative meta-analysis was not feasible. No formal protocol registration or standardized risk-of-bias assessment was performed. Instead, methodological limitations, assay-related uncertainties, and differences between experimental and clinical evidence were considered qualitatively during data interpretation. The aim was to provide an integrative and critical overview of current knowledge, identify consistent mechanistic themes, and highlight unresolved questions relevant to future research.

## 10. Conclusions

GRP, also known as UCMA, has emerged as a multifunctional vitamin K-dependent protein involved in the regulation of ectopic calcification and inflammation. The available evidence indicates that GRP plays an important modulatory role at the interface of mineral metabolism, inflammatory signaling, and tissue remodeling across both skeletal and extraskeletal tissues.

Mechanistically, GRP exerts anti-calcific effects through its exceptional calcium-binding capacity, which critically depends on its γ-carboxylation status. Carboxylated GRP appears to inhibit mineral deposition both directly and indirectly through regulation of extracellular vesicles and calciprotein particles, whereas undercarboxylated GRP predominates at sites of pathological calcification and reflects impaired vitamin K-dependent activation. Beyond calcification control, GRP also displays anti-inflammatory properties that appear to be, at least in part, independent of full γ-carboxylation, highlighting its dual functional role in chronic inflammatory and calcification-associated conditions.

Clinically, altered GRP levels and carboxylation patterns have been associated with chronic kidney disease, vascular and valvular calcification, osteoarthritis, and tumor-associated microcalcifications. Among these settings, the strongest clinical signal currently comes from chronic kidney disease, where reduced circulating GRP levels are linked to worsening renal function, disturbed mineral metabolism, vascular calcification, and cardiovascular risk. However, current evidence remains largely associative, and a direct causal role of GRP in disease progression has not yet been established.

Several important knowledge gaps remain. Standardized and clinically validated assays capable of reliably distinguishing circulating carboxylated and undercarboxylated GRP are still lacking, the relative contributions of circulating versus tissue-bound GRP pools remain insufficiently understood, and longitudinal data linking GRP dynamics to clinical outcomes are scarce. Addressing these limitations will be essential for clarifying the translational relevance of GRP.

In summary, GRP represents a promising integrative marker positioned at the crossroads of mineral metabolism, inflammation, and pathological calcification. Future studies aimed at elucidating its regulatory mechanisms, validating its biomarker potential, and defining its relationship with vitamin K status may improve mechanistic understanding and support more refined risk stratification in calcification-prone disorders.

## Figures and Tables

**Table 1:** Biochemical Pathways and Clinical Implications of Gla-Rich Protein GRP.

Clinical Context	Molecular Pathway Involving GRP	Downstream Biological Effects	Clinical Implications	References
Vascular Calcification	Carboxylated GRP binds calcium phosphate crystals via gamma carboxyglutamate residues. Forms complexes with MGP and fetuin-A. Regulates mineral maturation in extracellular vesicles and calciprotein particles.	Inhibition of hydroxyapatite nucleation and growth. Stabilization of extracellular particles. Suppression of osteogenic transdifferentiation of vascular smooth muscle cells.	Reduced GRP or predominance of undercarboxylated GRP associated with increased calcification burden and arterial stiffness.	[15,20,28,29,30,32,33]
Chronic Kidney Disease	Decline in renal function leads to reduced circulating GRP and altered loading of GRP into calciprotein particles and extracellular vesicles. Phosphate overload promotes VSMC osteogenic transition.	Impaired mineral buffering capacity. Enhanced inflammatory signaling and vascular calcification.	Circulating GRP inversely correlates with eGFR and vascular calcification. Potential early biomarker of cardiovascular risk in CKD MBD.	[13,15,42,104]
Inflammation and Immune Modulation	GRP expressed in monocytes and macrophages. Suppresses NF kappa B signaling and reduces TNF alpha, IL 1 beta, and PGE 2 production. Effects partly independent of carboxylation status.	Attenuation of pro-inflammatory cytokine release. Reduced crystal-induced macrophage activation.	Links inflammation and calcification axis. May reduce inflammatory burden in chronic diseases.	[23,30,34,35,70,71,72]
Calcific Aortic Valve Disease	Accumulation of undercarboxylated GRP at mineral deposition sites. Limited recruitment of carboxylated GRP to mineral-bound protein complexes.	Reduced local inhibition of crystal growth. Valvular interstitial cell osteogenic activation.	Suggests vitamin K-dependent dysfunction contributes to valvular calcification. GRP may reflect local mineral imbalance.	[20,23,81,82,83,84,85,86,87,88,89,90,91]
Osteoarthritis	GRP modulates cross talk between calcification and inflammation in chondrocytes and synoviocytes. Inhibits matrix metalloproteinase 13 and COX 2 expression. Inhibits ADAMTS-mediated aggrecan degradation.	Reduced extracellular matrix breakdown. Decreased inflammatory mediator production. Control of basic calcium phosphate crystal-induced inflammation.	Protective role in joint degeneration. *UCMA* deficiency is associated with worsened cartilage damage.	[34,41,68,98,99]
Osteogenic Differentiation	GRP regulated by Runx2 and Osterix. Modulates expression of osteogenic markers such as osteocalcin and osteopontin. In vascular cells, upregulates alpha-smooth muscle actin and suppresses osteogenic genes.	Context-dependent control of osteoblast maturation and phenotypic switching.	Suggests modulatory role in skeletal and ectopic bone formation. Not essential but regulatory.	[20,30,39,40,73,74,75,76,77]
Tumor-Associated Microcalcifications	Predominance of undercarboxylated GRP in tumor microcalcifications. Altered vitamin K-dependent carboxylation within tumor microenvironment.	Reduced calcification-inhibitory function. Association with pathological mineral deposition in malignant tissues.	GRP may represent a vitamin K-responsive component of tumor calcification biology.	[23,30,38,100,101,102,103]
Vitamin K Deficiency or Antagonism	Warfarin or low vitamin K reduces gamma carboxylation of GRP. Functional inactivation of calcification-inhibitory capacity.	Accelerated medial vascular calcification in experimental models.	Highlights importance of vitamin K status in GRP-mediated vascular protection.	[6,8,13,25,54,55,56,57,58,104]

## Data Availability

No new data was created or analyzed in this study. Data sharing is not applicable to this article.

## Abbreviations

The following abbreviations are used in this manuscript:

ADAMTS	A disintegrin and metalloproteinase with thrombospondin motifs
α-SMA	Alpha-smooth muscle actin
ApoE	Apolipoprotein E
BMP-2	Bone morphogenetic protein 2
CAVD	Calcific aortic valve disease
cGRP	Carboxylated Gla-rich protein
CKD	Chronic kidney disease
CKD-MBD	Chronic kidney disease–mineral and bone disorder
COL2	Collagen type II
COL10	Collagen type X
COX-2	Cyclooxygenase-2
CPP(s)	Calciprotein particle(s)
CVD	Cardiovascular disease
eGFR	Estimated glomerular filtration rate
ETS	E26 transformation-specific transcription factor family
EV(s)	Extracellular vesicle(s)
FGF-23	Fibroblast growth factor 23
Gla	γ-Carboxyglutamate
GRP	Gla-rich protein
IL-1β	Interleukin-1 beta
LPS	Lipopolysaccharide
MEF2	Myocyte enhancer factor 2
MGP	Matrix Gla protein
MMP-13	Matrix metalloproteinase-13
Msx2	Msh homeobox 2
NF-κB	Nuclear factor kappa B
OA	Osteoarthritis
OC	Osteocalcin
OPG	Osteoprotegerin
OPN	Osteopontin
Osx	Osterix
PGE-2	Prostaglandin E2
Runx2	Runt-related transcription factor 2
Sox9	SRY-box transcription factor 9
STAT1	Signal transducer and activator of transcription 1
TGF-β1	Transforming growth factor beta 1
THP-1	Human monocytic leukemia cell line
TNF-α	Tumor necrosis factor alpha
ucGRP	Undercarboxylated Gla-rich protein
*UCMA*	Upper zone of growth plate and cartilage matrix-associated protein
VC	Vascular calcification
VIC(s)	Valvular interstitial cell(s)
VK	Vitamin K
VKDP(s)	Vitamin K-dependent protein(s)
VSMC(s)	Vascular smooth muscle cell(s)
